# Satellite Edge Computing for the Internet of Things in Aerospace

**DOI:** 10.3390/s19204375

**Published:** 2019-10-10

**Authors:** Yuxuan Wang, Jun Yang, Xiye Guo, Zhi Qu

**Affiliations:** College of Intelligence Science and Technology, The National University of Defense Technology, Changsha 410073, China

**Keywords:** edge computing, internet of things, intelligent satellite

## Abstract

As one of the information industry’s future development directions, the Internet of Things (IoT) has been widely used. In order to reduce the pressure on the network caused by the long distance between the processing platform and the terminal, edge computing provides a new paradigm for IoT applications. In many scenarios, the IoT devices are distributed in remote areas or extreme terrain and cannot be accessed directly through the terrestrial network, and data transmission can only be achieved via satellite. However, traditional satellites are highly customized, and on-board resources are designed for specific applications rather than universal computing. Therefore, we propose to transform the traditional satellite into a space edge computing node. It can dynamically load software in orbit, flexibly share on-board resources, and provide services coordinated with the cloud. The corresponding hardware structure and software architecture of the satellite is presented. Through the modeling analysis and simulation experiments of the application scenarios, the results show that the space edge computing system takes less time and consumes less energy than the traditional satellite constellation. The quality of service is mainly related to the number of satellites, satellite performance, and task offloading strategy.

## 1. Introduction

In recent years, relying on the construction of infrastructure (such as the terrestrial Internet and mobile network) and the popularization of smart devices, IoT technology has developed rapidly [1]. Iot technology has made excellent progress in many application scenarios, such as healthcare [2,3], transportation [4], agriculture [5], smart cities [6], smart homes [7], environment monitoring [8], etc. The impact of the IoT on the Internet and economy is impressive. Some anticipate that there will be as many as 27 billion connected IoT devices by 2025 [9]. However, the terrestrial network has limited coverage and is mainly concentrated in urban areas. For some harsh environments, such as deserts, forests, mountains, and oceans, the terrestrial network cannot be covered entirely. Additionally, in the face of natural disasters, such as floods, earthquakes, tsunamis, etc., the terrestrial network is vulnerable. With its extensive coverage and strong system invulnerability, satellite communication systems can provide access services for IoT terminals in remote areas, realizing the “Internet of Everything” in the real sense of the world. There are several large satellite network providers, such as Intelsat, Iridium, GlobalStar, Teledesic, etc. [10]. With the development of microsatellite technology, the cost of satellite development and deployment has decreased significantly. LEO satellite constellations have begun to provide powerful support for IoT devices. The global market for satellite IoT services reached 1.7 billion dollars in 2017 [9].

In the current satellite IoT system, the terminal forwards the acquired data to the terrestrial cloud platform through satellites for post-processing. However, cloud computing platforms are often physically and logically distant from the terminal. It results in a high communication latency between the terminal and the cloud [11]. With the coming of the age of Big Data, the amount of data has exploded. According to Cisco’s 2017 NVI report, globe IoT data reached 2 EB/month in 2016 and is expected to reached 14 EB/month by 2021, with a compound annual growth rate of 49% [12]. The increasing amount of data brings in substantial stress on the network links. The situation will be even worse as satellites are limited by orbits and onboard resources.

In order to help address these issues, the concept of edge computing has been used in satellite IoT systems. The basic principle is to extend cloud platform capabilities to the edge of the network [13]. It supports data processing via their shared pool of computing resources, minimizing the amount of data sent to the cloud for faster processing results [14]. The combination has the following three key benefits: (1) lowering the amount of communication data and reducing the bandwidth demands on network link; (2) reducing the latency for application and services; and (3) support mobility of devices and geographically-distributed applications.

Traditional satellites are highly customized according to specific functions. The satellite functions have been identified at the beginning of the design and cannot be changed during the run-time. Moreover, the on-board resource is designed for specific applications, rather than the universal computing that supports multiple applications. It is challenging to share local resources with others. Therefore, edge computing cannot be directly applied to traditional satellites. Combined with the mobile edge computing and the characteristics of the satellite itself, this paper proposes an intelligent satellite (iSat) suitable for satellite edge computing. iSat is a class of multi-purpose satellite with a powerful standardized hardware platform and a fault-tolerant expandable satellite operation system. It can load different apps and share on-board resources with other satellites on-demand, providing a more robust and flexible personalized space service.

The main contributions of this article are twofold. First, we design hardware structure and software architecture of iSat to transform satellites from traditional relay nodes into space edge computing nodes. It can dynamically load software in orbit, flexibly share on-board resources, and provide services coordinated with the cloud. Second, based on the mobile edge computing, we combine the mobile edge computing and satellite orbit to establish a system model of the application scenario and analyze the execution time and energy consumption of the task. Simulation experiment results show that the space edge computing system takes less time and consumes less energy than the traditional satellite constellation.

The remainder of this paper is organized as follows: First, a rapid overview of relevant related work is presented in Section 2. Then, the architecture and application scenarios of iSat are proposed in Section 3. Next, the system model of the space edge computing system is analyzed in Section 4, which consists of the computation model, communication model, and satellite execution model. In Section 5, the simulation results are presented and discussed. Finally, Section 6 concludes with an exploration of future works.

## 2. Related Works

In this section, we first describe some of the existing and planned satellite constellations for IoT. At present, satellites are widely recognized as an essential part of the IoT. They form communication satellite constellation, which realizes applications in data acquisition, system monitoring, tracking and positioning, and message transmission. Next, some new concept satellites proposed in recent years, and their characteristics are summarized. These new concept satellites represent some of the current trends in satellites.

### 2.1. Satellite Constellation for IoT

The typical satellite system that has been built for IoT is Orbcomm, ARGOS, LoRaWAN, etc.

Orbcomm is a commercial global low-orbit satellite communication system. The system uses packet switching to achieve bidirectional short data transmission, providing global low-rate wireless data communication services [15]. It consists of 47 satellites, each of which covers a surface with a radius of 5100 km [16]. With the Orbcomm satellite communication system, users can carry out applications, including remote data collection, system monitoring, tracking and positioning of vehicle and mobile facilities, the transmission of short messages, sending and receiving emails, etc.

ARGOS (the Advanced Research and Global Observation Satellite) is a satellite communication system for data collection and positioning established by France and the United States. The system uses satellites to transmit various environmental monitoring data and locates the carrier of the measuring instrument. It provides an excellent means of communication for hydrological and meteorological monitoring instruments at high latitudes [17]. ARGOS is a typical space IoT system that uses satellite networks to interconnect people, platforms, and sensors. It can quickly, accurately, and extensively collect water temperature and salinity profiles from 0 to 2000 m in the global ocean. It helps to understand the real-time changes of the ocean in a more detailed way, improve the accuracy of climate and ocean forecasting, and effectively defend against the threats posed by the increasingly severe global climate and marine disasters.

Inmarsat Plc and LPWAN equipment manufacturer Actility jointly developed the LoRaWAN-based IoT in January 2017. The LoRaWAN network is the world’s first global IoT network. With a backbone of terrestrial and satellite networks, the Inmarsat LoRaWAN network can deliver on its strategy to bring the IoT to every corner of the globe. At present, its early applications cover asset tracking, agribusiness, and oil and gas.

Other planned satellite IoT systems are described as follows. Australian space technology company Fleet plans to launch 100 nanosatellites between 2018 and 2020. The US communications company Kepler plans to complete the deployment of a space network consisting of 140 Ku-band nanosatellites in 2022. HeliosWire plans to launch 30 satellites to build a space IoT, supporting 5 billion sensors with 30 MHz bandwidth. Russia’s SPUTNIX plans to deploy about 200 IoT satellites in low Earth orbit in 2025.

These IoT satellites can provide data communication services for sensors or ground equipment. They collect data from ground devices and forward them to the terrestrial cloud. However, the capability of processing in-orbit is weak. It will result in a large amount of communication data and a high latency.

### 2.2. New Concept Satellite Research

Traditional satellites can be divided into two parts: platform and payload. The payload determines the function of the satellite. The platform provides relevant guarantee services for the payload. With the development of microsatellite technology, various new technologies are continuously applied to satellites, such as integrated circuits, digital signal processing, MEMS, and additive manufacturing, etc. Various countries are actively exploring the next-generation satellite. Many new concept satellites have emerged. These new concept satellites mainly focus on the following two aspects: module standardization and function software definition.

In terms of improving the modularization level of satellites, some institutions carry out the relevant research work and have achieved some results. In the standardization of satellite structure, a low-cost spacecraft platform with multi-payload adaptability for low-orbit space missions, called CubeSat, is proposed [18]. It uses a cube with a side length of 10 cm as a standard unit and can flexibly combine according to task requirements. In the standardization of the satellite interface between hardware modules, a plug-and-play satellite (PnPSat) is proposed [19]. Its components are analogous to the USB components of a personal computer, which can be formed by simply plugging together. In terms of improving on-board resource sharing, a satellite is divided into a standard set of subsystems, called Satlets [20], which can “sense” they are aggregated and cooperate in sharing resources. These proposed satellites have promoted the standardization of satellite modules, the plug-and-play of devices, and the enhancement of satellite resource sharing capabilities. However, the application of these satellites is still the traditional model. To improve the capability of satellite service in-orbit, some institutions conducted related studies on satellite software.

The software-defined satellite is inspired by software-defined radio (SDR). In 2012, the International Space Station carried out three SDR payload for technical verification. The “Eutelsat-quantum”, designed by European Space Agency (ESA) and Eutelsat, is a software-defined payload satellite [21]. It changes the parameters of the communication payload by software defined to achieve functional reconfiguration. The satellite is expected to launch and conduct in-orbit experiments in 2019. The Institute of Software, Chinese Academy of Sciences (ISCAS) organized the Software Defined Satellite Technology Alliance in 2017. The alliance aims to create an open-source platform-level software solutions for satellites using a common computing platform, creating the conditions for flexible software definition and expansion of satellite capabilities. The first experimental satellite “Tianzhi-1” for verifying the technology was launched in November 2018. Currently, although some satellites already have a certain degree of software definition capabilities, the related technology of software defined satellites is not mature enough. However, such satellites have enormous potential for various applications. Moreover, some studies have combined satellites with edge computing to deploy edge computing servers on satellites for lower latency and boarder coverage of terrestrial–satellite communication [22,23]. However, they pay attention to the performance of terrestrial–satellite communication, and neither of them considered the effects of satellite orbits. To improve the QoS of IoT system, we propose the iSat for IoT in aerospace. To the best of author’s knowledge, it is the first satellite edge computing for IoT. iSat not only has software-defined capabilities but also can perform edge computing, such as onboard resource virtualization, resource on-demand scheduling, task offloading, and cloud–edge collaboration.

## 3. The Architecture and Application Scenarios of Satellite Edge Computing

**Figure 1:**
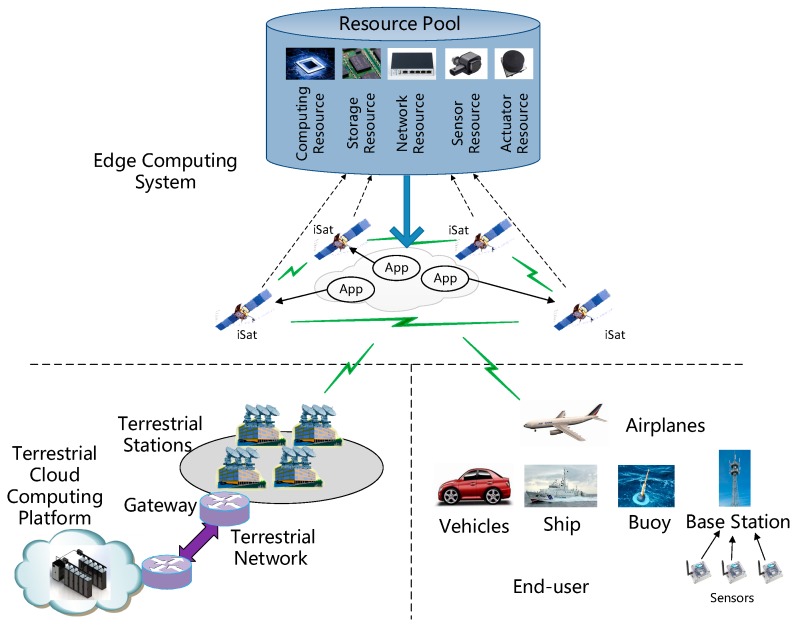
The architecture and application of satellite edge computing.

We propose the architecture of the space edge computing system and its application scenarios, as shown in Figure 1. Several iSats form a space edge computing system, which can acquire, store, process, and forward data from the end-users (such as vehicles, airplanes, ships, buoys, and sensors). For small sensors, data can be relayed to the satellite via the base station. In the face of different end-users, the space edge computing system can flexibly change the applications on each iSat to satisfy different mission requirements. Additionally, iSats can virtualize on-board resources and form a resource pool. Depending on the task, the space edge computing system can allocate resources on demand. The detailed structure and application scenarios are illustrated as follows.

### 3.1. iSat System

The space edge computing system consists of several iSats. iSat, as an edge computing node, uses a powerful standardized hardware platform and a fault-tolerant and expandable satellite operating system. It can load different apps according to the task requirements, providing customized services. The capabilities of iSat include and are not limited to the following:

1. Provide a consistent operating paradigm across multiple satellite infrastructures.
2. Support large-scale, distributed space network environments.
3. Support application integration, orchestration, and migration.
4. Meet hardware resource limits and cost constraints.
5. Capable of running on confined and unstable networks.
6. Meet the needs of ultra-low latency applications.
7. On-board resources can be flexibly shared with multiple users or applications.

The difference between iSat and traditional satellites will be illustrated from both hardware and software.

#### 3.1.1. Hardware Structure

In terms of hardware structure, traditional satellites usually consist of two parts: platform and payload. The platform is responsible for satellite management, including attitude determination and control (ADCS), communication (Comm), electrical power (EPS), telemetry and telecontrol (TM and TC), thermal control (TCS), etc.

The payloads perform specific tasks with the support of the platform. However, this structure leads to the independence of each subsystem and a complicated interface relationship. There are several problems in the following aspects:

1. Low system integration. Each subsystem has substantial weight, high power consumption, large volume, and complicated interfaces. The optimization design is limited to the subsystem level. The overall performance cannot be guaranteed to be optimal.
2. Low resource utilization. There are a lot of redundant designs in each subsystem, but these idle resources cannot be effectively utilized.
3. Low bus bandwidth. The data transmission capability between subsystems is limited. It is impossible to achieve effective coordination and integrated design between systems.
4. Low reusability. Each subsystem needs to be customized, resulting in poor compatibility and interchangeability. The development and expansion of the system are costly.

**Figure 2:**
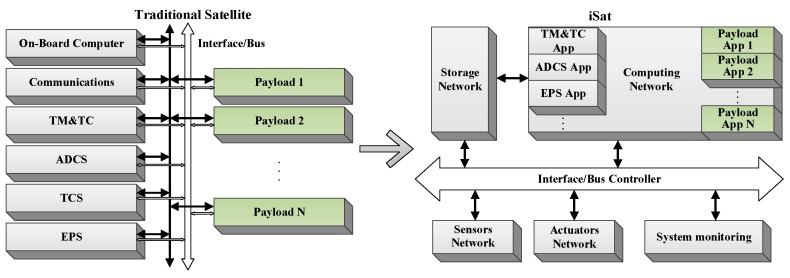
The hardware structure of the traditional satellite and iSat.

In order to make the most use of the resources on the satellite, iSat changes the hardware structure of the traditional satellite. It uses an integrated structure to realize the re-division of the function and structure of the satellite platform and payload. It combines the same or similar parts of each subsystem. The internal data interaction is achieved via a standardized bus. The comparison between the two structures is shown in Figure 2.

The hardware structure of iSat consists of computing network, storage network, interface/bus controller, system monitoring module, sensors network, and actuators network. The computing network is a distributed heterogeneous computing platform, which is the core of iSat. The computing network not only needs to complete the computing tasks of traditional satellite platforms but also needs to complete the general calculation function of various payloads. The storage network adopts a distributed structure to realize the storage of various types of information and software to maximize the utilization of limited storage resources. The interface/bus controller provides standardized data interfaces for different devices. The system monitoring module monitors all essential parameters to ensure the regular operation of the satellite. When there is a problem with the satellite, a reset signal is given in time to help the satellite restart. During the restarting, the system monitoring module will maintain the basic parameters of the satellite, such as attitude and temperature, etc. The sensors network contains all the sensors in the traditional satellite platform and payload, such as magnetometers, IMUs, star sensors, cameras, etc. Through the sensor network, it is possible to collect the information for satellite operation, monitor the operating status, and facilitate uniform processing analysis. The actuator network includes the actuators required to perform a series of tasks such as satellite attitude control, orbital change, and payload adjustment.

#### 3.1.2. Software Architecture

**Figure 3:**
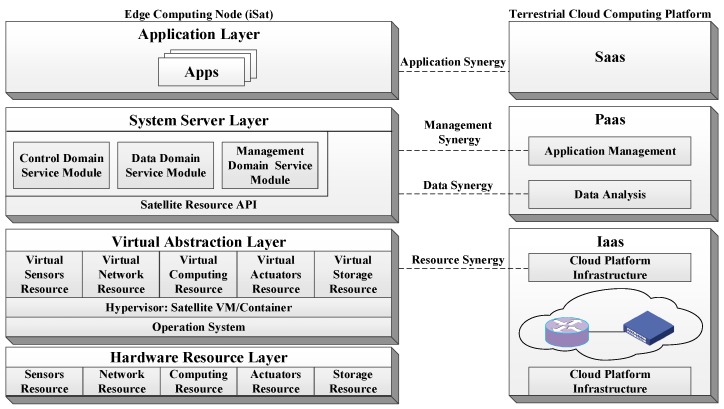
The software architecture and collaborative mode of iSat.

As an intelligent node for space edge computing, the core of iSat is the software. Unlike traditional satellites, the functionality of iSat can be defined in-orbit by loading apps. iSat cannot only operate separately to provide a variety of services but also form a space edge computing system through inter-satellite links. The software architecture and collaboration mode of iSat are shown in Figure 3.

Unlike the tight coupling of traditional satellite software and hardware, the iSat satellite software uses an open hierarchical architecture. Each layer corresponds to the function of the terrestrial cloud computing platform, which facilitates the cloud-edge collaboration to provide services. The functions of each layer and the collaboration mode will be described in detail below.

The hardware resource layer contains all the hardware units of the iSat. According to the hardware structure, all the devices will be abstracted into five categories of resources: computing resources, network resources, sensors resources, actuators resources, and storage resources.

The virtual abstraction layer integrates various resources through resource virtualization technology to achieve unified management. Although there is currently no resource virtualization method for satellites, the virtualization methods for the cloud platform, such as virtual machines or containers, have been used in embedded systems [24,25]. These technologies will be used to implement the virtualized packaging of onboard devices. The virtualized satellite resources can be coordinated with the resources of the terrestrial cloud computing platform. By receiving and executing resource management strategies of the cloud, on-board resources can be deployed on demand.

The system service layer provides APIs for calling on-board resources and three specific service modules, including control domain service module, data domain service module, and management domain service module. The control domain service module provides real-time services, such as perception of the environment, real-time communication, satellite control system execution, and device resource management. The data domain service module provides preprocessing operations for data. It reduces communication bandwidth by filtering useless data. It includes stream data analysis, video and image analysis, intelligent computing, and data mining. Through the module, satellites can implement data synergy with cloud computing platforms. The cloud develops a data transmission and execution strategy of satellite and delivers it to satellite for execution. The cloud provides storage, analysis, and value mining of the massive received data. The management domain service module provides optimized management strategies, including resources, services, and data. Satellites can implement management synergy with cloud computing platforms via the module. The satellite provides an application runtime environment and management APIs. The cloud realizes the management of the entire life cycle of satellite applications.

The application layer contains various satellite apps. Satellites can run different apps to provide the corresponding service capabilities based on the needs of the cloud. The cloud needs to provide a corresponding deployment strategy for different satellites according to user requirements.

#### 3.1.3. Uploading Apps

Apps, as the soul of iSat, can be uploaded according to demands. According to the characteristic of satellite applications, apps can be divided into two types based on CPU and FPGA.

**Figure 4:**
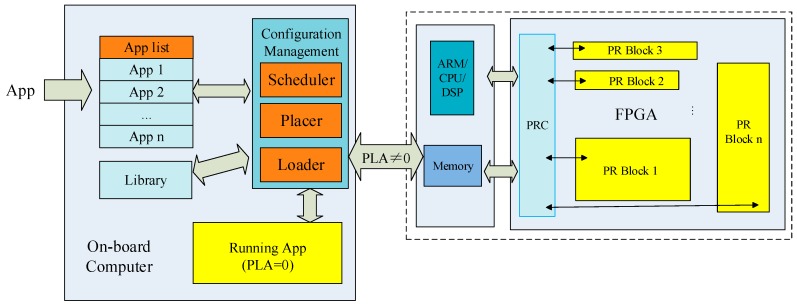
The on-board apps configuration module.

It needs two parts to upload apps, i.e., terrestrial-satellite communication module and on-board apps reconfiguration module. The terrestrial monitor station will send uploading instruction, the file of apps and related configuration file to satellite by terrestrial-satellite communication module. The on-board apps configuration module can support uploading, verification, registration, running and management of apps. The module is designed as shown in Figure 4.

The module consists of three parts: app list, library, and configuration management. Each iSat has an app list that is a collection of all apps for the satellite. The app list can be represented by the following expression: *S_i_* = Σ[App_i_(*PS_i_*,*MS_i_*,*PLA_i_*,*at_i_*,*et_i_*,*dt_i_*)], where *S_i_* denotes the app list of iSat *i*, App*_i_* denotes the app *i*, *PS_i_* denotes the process scale of app *i*, *MS_i_* denotes the memory scale of app *i*, *PLA_i_* denotes the programable logic area, *at_i_* denotes the arrival time of app *i*, *et_i_* denotes the end time of app *i*, and dti denotes the duration time of app *i*. apps can be pre-configured or uploaded by the ground monitor station after the satellite enters the orbit. According to the relevant description of all apps of the iSat, a task scheduling module sorts and schedules the apps to obtain an execution queue. Then the loading and running of the two types of apps through the placer and loader.

The library is used to store validated executable files and bitstream files. There are two purposes for designing the library. One is to improve the reliability of application uploading. Satellite orbiting will cause terrestrial–satellite communication to be intermittent. The apps before storage will be verified to avoid damage to the satellite caused by incomplete and incorrect files. The other is to reduce the dependence of satellites on the ground monitor system. Upon losing connection to the ground, iSat can load apps on demand from the library.

For iSat application App*_i_*, there is a separate process corresponding to it. According to the value of the *PLA_i_* in App*_i_*, the running environment to which the task loader loads the task is determined. The loader will load the app to the appropriate running environment, i.e., when *PLA_i_* is equal to zero, the app can be run directly on the satellite operating system; when *PLA_i_* is not equal to zero, the app needs to be placed into the reconfigurable FPGA resources.

The on-board app reconfiguration module is managed by a house-keeping system to increase system reliability and autonomy.

### 3.2. Application Scenarios

According to [22], the typical satellite constellation-based IoT application scenarios are divided into two groups: delay-tolerant applications (DTAs) and delay-sensitive applications (DSAs). We will describe the capabilities of iSat in these two application scenarios.

For DTA, the satellites provide automatic store-and-forward data communication services in networks [26]. In general, these applications have a characteristic feature of frequent and prolonged temporary disconnections and long propagation delays. A typical application is the ARGO program for global ocean observations using buoys. Considering that the terrestrial system cannot cope with ocean monitoring, the use of satellites could be irreplaceable. Due to the constraints of the orbit, satellites can only provide services in some arcs. Additionally, traditional satellites mainly provide store-and-forward data communication services, which are insufficient for data processing. iSat can load applications in orbit, provide other services, such as data processing in idle arcs, or share its idle resources with other satellites. Thus, as an intelligent node of space edge computing, iSat can provide services for user-ends of different locations, different communication protocols, and different processing algorithms. It improves the resource utilization and service efficiency of the entire space system.

DSA is an entirely different scenario with strict requirements for low latency and high reliability. In such scenarios, satellite missions are usually initiated by ground control stations. All kinds of information obtained by the satellites are forwarded to the ground for unified processing. This leads to a significant increase in link latency. iSats constitute a space edge computing system, which realizes inter-satellite data interconnection and resource sharing through inter-satellite links. The tasks can be offloaded to other iSats for collaborative processing. This will reduce the amount of data transmission between satellites and ground stations and provide more efficient services.

## 4. System Model

In this section, we introduce the system model of space edge computing, which includes the computation model, the communication model, and the satellite execution model.

### 4.1. Computation Model

Firstly, the computation model will be illustrated. We assume that the system contains *N* terrestrial user terminals, *M* iSats as nodes of space edge computing, and a ground station as a terrestrial cloud computing platform. For terrestrial user terminals, each terminal can execute an application and generate a series of homogeneous service requests. So, the task *i* of the user terminal can present as:

(1) $$Task_{i}=\left\{c_{i},d_{i},P_{i}\right\}$$

where *c_i_* represents the number of computing resources required to accomplish the task; for example, *c_i_* can be quantified by the number of CPU cycles. The variable *d_i_* denotes the size of the computation input file describing some information of the task, such as the program codes or the corresponding data, and *P_i_* represents the probability of the task being offloaded to the cloud. We assume that each satellite has the same computing power *CP_i_* (for example CPU cycles/s). Therefore, the time required for the satellite to process the task *i* is *T_SC_*:

(2) $$T_{SC}=\frac{c_{i}}{CP_{i}}$$

It is assumed that the resources of the terrestrial cloud computing platform are large enough, so the computation time for the task is negligible.

For the energy consumption of satellites, it is proportional to the square of the frequency of the CPU [27,28]. Thus, we have:

(3) $$E_{i,computation}=\kappa CP_{i}^{2}c_{i}$$

where *κ* is the effective switched capacitance, which depends on the chip architecture [29].

### 4.2. Communication Model

Next, the communication model for wireless access in space edge computing will be introduced. For terrestrial terminals, it sends all tasks to the satellite for processing. If the satellite resources are sufficient, the mission is performed on the satellite; otherwise, the satellite will further offload the mission to the terrestrial cloud computing platform.

Therefore, the communication links include ground terminals transmitting tasks to satellites, satellites offloading tasks to terrestrial cloud computing platforms, terrestrial cloud computing platforms transmitting results to satellites and satellites reporting results to terrestrial terminals. According to the research in [27], the transmission time consumption from the cloud computing platform to the satellite and the satellite to the terminal is neglected in this work, due to the face that the size of the computation outcome data is much smaller than that of the computation input data. Only the communication links from terminal to satellite and satellite to ground stations will be analyzed.

Considering the mutual interference between the terminals and background noise, the uplink data rate for terminal *t* can be calculated as follows [30]:

(4) $$R_{Ti2S}=W_{Ti2S}\log_{2}\left(1+\frac{p_{t}g_{t,s}}{n_{0}+\sum_{j\in N,j\neq i}p_{j}g_{j,s}}\right)$$

where *W_Ti_*_2*S*_ is the channel bandwidth, and *p_t_* is terminal *t*’s transmission power, which is determined by the satellite. Besides, *g_t,s_* is the channel gain between the terminal *t* and the satellite *s*, and *n*_0_ denotes the background noise power. According to Equation (4), the transmission time of task *i* generated by terminal *t* to the satellite can be calculated as follows:

(5) $$T_{i,upload}=\frac{d_{i}}{R_{Ti2S}}+\frac{D_{T2S}}{c}$$

where *D_T_*_2*S*_ is the distance between the terminal that generates the task *i* and satellite in communication, and *c* is the speed of light.

Then, the energy consumed by the satellite receiving data is described as follow:

(6) $$E_{i,receive}=p_{sr}\frac{d_{i}}{R_{Ti2S}}$$

where *p_sr_* is the satellite receiving power. Similarly, the transmission rate between satellite *s* and ground station can be described as follow:

(7) $$R_{S2C}=W_{S2C}\log_{2}\left(1+\frac{p_{s}g_{s,c}}{n_{0}+\sum_{j\in SA,j\neq s}p_{j}g_{j,c}}\right)$$

where *SA* is a set of satellites that can communicate with the ground station. Other parameters are like Equation (3). The transmission time of satellite *s* for offloading the task is described as follows:

(8) $$T_{i,offload}=\frac{d_{i}}{R_{S2C}}+\frac{D_{S2C}}{c}$$

The energy consumed by the satellite transmitting data is described as follow:

(9) $$E_{i,transmit}=p_{s}\frac{d_{i}}{R_{S2C}}$$

### 4.3. Satellite Orbit Model

Unlike many previous studies in mobile edge computing, a satellite cannot always communicate with a terminal and the cloud computing platform. Therefore, the latency of waiting for a satellite connection needs to be considered during mission execution.

**Figure 5:**
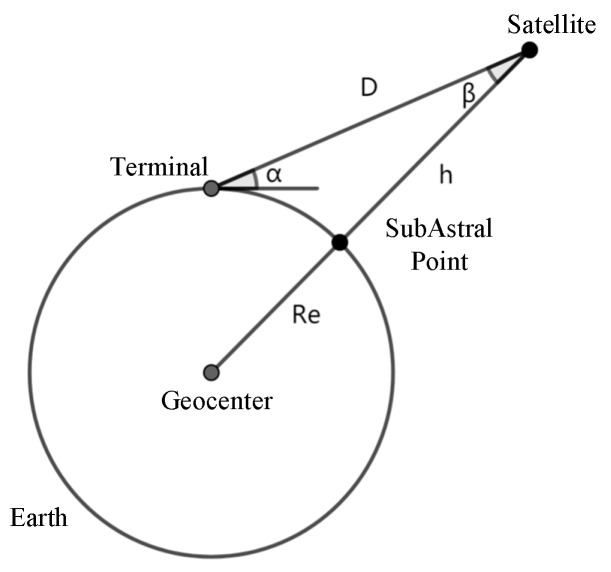
The space geometry of a communication link between a satellite and a terminal.

The space geometry of the link between the satellite and a fixed location on the ground is shown in Figure 5.

Here, *α* is the elevation angle of the mobile device, *β* is the half-angle of view of the satellite, *R_e_* is the radius of the earth, and *h* is the altitude of the satellite. Data transmission is only possible when the satellite is in the window of the mobile device. Regardless of the influence of other factors, when *α* > 0, the satellite is in the communication windows. According to the geometric relationship, the expression of *α* can be calculated as follows:

(10) $$\alpha =\arctan\frac{\cos\Delta \lambda \cos\varphi_{t}\cos\varphi_{s}+\sin\varphi_{t}\sin\varphi_{s}-\frac{R_{e}}{R_{e}+h}}{\sqrt{1-{\left(\sin\varphi_{t}\sin\varphi_{s}+\cos\Delta \lambda \cos\varphi_{t}\cos\varphi_{s}\right)}^{2}}}$$

Here, Δ*λ* = *λ_t_* − *λ_s_*, *λ_t_* and *φ_t_* are longitude and latitude of the terrestrial terminal, respectively. *λ_s_* and *φ_s_* are longitude and latitude of the satellite, respectively.

### 4.4. Time and Energy Consumption

We assume that the process by which the ground terminal generates a task is a Poisson process with a parameter of *λ*. *λ* denotes the average speed of the task generation. Since a ground terminal cannot always communicate with a satellite, it is necessary to wait for the satellite to move into the window. We assume that the waiting time is *T_i,wati_upload_*. Next, it needs to be classified according to whether the task will be offloaded to the cloud computing platform.

(1) Edge computing: According to Equation (2), it takes *T_SC_* for the satellite to accomplish task *i*. Currently, it is necessary to re-determine the visibility of the satellite *s* and the terminal *t*. If visible, the results can be returned immediately. If it is not visible, regardless of results passing between satellites, the satellite needs to wait for the next communication window to send the result back. We assume the waiting time is *T_i,wati_return_*. Therefore, the total time for task execution in edge computing is:

(11) $$T_{i,edge}=T_{i,wait\_upload}+T_{i,upload}+T_{SC}+T_{i,wait\_return}$$

Satellite energy consumption consists of three parts, the energy consumed by the satellite to maintain its operation, the energy consumed by the satellite to receive data, and the energy consumed by the satellite processing mission. Therefore, the total energy consumption for task execution in edge computing is:

(12) $$E_{i,edge}=E_{i,receive}+E_{i,computation}+E_{i,static}$$

where *E_i,static_* is the energy consumed by the satellite to maintain its operation, assumed to be a fixed value.

(2) Cloud computing: Similarly, to offload tasks to the cloud computing platform, the satellites need to move into the ground station window. We assume the waiting time is *T_i,wait_offload_*. Considering that the computing power of the cloud computing platform is large enough, and the size of the outcome is quite small, the processing time of the cloud computing platform and the time that the cloud computing platform feeds back to the satellite are neglected. Therefore, the total time for task execution in cloud computing is:

(13) $$T_{i,cloud}=T_{i,wait\_upload}+T_{i,upload}+T_{i,wait\_offload}+T_{i,offload}+T_{i,wait\_return}$$

The total energy consumption for task execution in cloud computing is:

(14) $$E_{i,cloud}=E_{i,receive}+E_{i,transmit}+E_{i,static}$$

According to Equations (11) and (13), the total time to complete all the tasks can be calculated as follows:

(15) $$T_{total}=\sum_{i}P_{i}T_{i,cloud}+(1-P_{i})T_{i,edge}$$

Similarly, according to Equations (12) and (14), the total energy consumption to complete all the tasks can be calculated as follows:

(16) $$E_{total}=\sum_{i}P_{i}E_{i,cloud}+(1-P_{i})E_{i,edge}$$

## 5. Performance Evaluation

In this section, we show illustrative results to demonstrate the performance of our proposed satellite edge computing system. We will compare the performance of satellite edge computing systems with traditional satellite constellations through two indicators: total time consumption and total energy consumption. The total time and total energy consumption to complete all the tasks are defined by Equations (15) and (16), respectively.

According to the following settings, the total time and total energy consumption of space edge computing and traditional satellite constellations are compared. The upstream rate is 10 Mbps, and the downstream rate is 100 Mbps. The transmitting power *p_s_* = 0.12 W. The size of end-user data for each task is a random number within 400 M. Additionally, the effective switched capacitance *κ* = 10^−28^, the CPU resource on satellite is 1 GHz, and the processing capacity is 1000 cycles/bit. These parameters are obtained by referring to [23,31]. The orbit of the Iridium satellite constellation is chosen as the simulated constellation orbit. The specific parameters are shown in Table 1. The experimental constellation consisting of 66 satellites is simulated by automatically building the Walker constellation in STK. Through STK, we obtain the satellite coordinates of the simulated satellite constellation throughout January 1, 2000. The sampling interval is 10 s.

The simulation of the specific work of the satellite is implemented in MATLAB. The latitude and longitude of the terrestrial cloud computing platform are fixed, and the latitude and longitude of the terrestrial user terminal are randomly generated. The tasks generated by each terminal follows a Poisson process. The average generation rate is *λ*. It is worth noting that most of the results of simulation studies in this section are based on an average over a few Monte Carlo simulations for various system parameters.

The service flow of traditional satellite is as follows:

Step 1: The terrestrial user terminal generates tasks.
Step 2: The terminal sends the task to the satellite.
Step 3: The satellite forwards the mission to the terrestrial station.
Step 4: The terrestrial station sends the results back to the satellite after processing.
Step 5: The satellite sends the result back to the terrestrial terminal.

The processing flow of the space edge computing system is as follows:

Step 1 and Step 2 are the same as the traditional satellite processing steps.
Step 3: iSat will choose whether to offload the task to the terrestrial cloud computing platform.
Step 4: If the task is executed on iSat, the results will be sent back to the terminal. If the task is offloaded to the terrestrial cloud computing platform, the result is sent back to the ground terminal via satellite after the task is executed.

**Figure 6:**
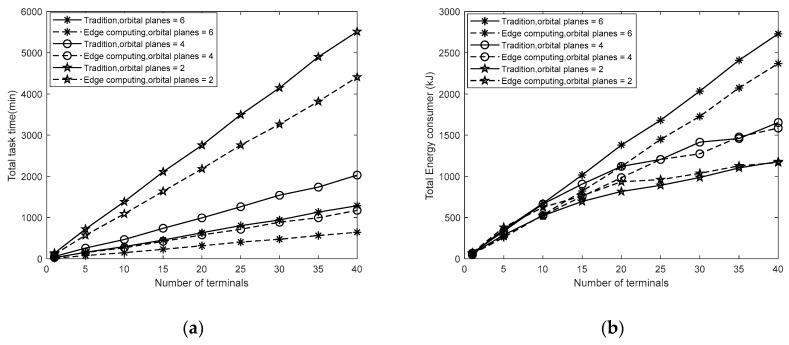
(**a**) the total time spent in terms of the number of terminals; (**b**) the total energy consumption in terms of the number of terminals.

Figure 6 evaluates the total time and energy for all tasks in terms of the number of terrestrial terminals, respectively. With the increase in terminals, the total time of tasks has increased almost linearly. The time spent in the traditional mode is much longer than that of the space edge computing. Additionally, we compared the performance of the number of satellites on the total time spent. In order to ensure basic ground coverage, we have directly reduced several orbital planes. As the number of satellites decreases, time-consuming increases rapidly. Especially when the number of satellite orbital plane is small, the time consumption will increase significantly. Although increasing the number of satellite planes helps to reduce the total task time, it will lead to an increase in the cost. For energy consumption, in general, as the number of terminals increases, the total energy consumption increases. As the number of orbit planes decreases, the total energy consumption decreases. This is because of the reduction in the number of satellites. When the number of satellite orbital planes is six and four, the energy consumption in the conventional mode is more significant than that in the edge computing mode. When the number of satellite orbital planes is two, the result is reversed. This is because when the number of satellites decreases, in the space edge computing mode, the energy consumed by a single satellite and the working time increase. Therefore, in order to obtain a better quality of service, the system needs to be optimized for the number of satellites.

**Figure 7:**
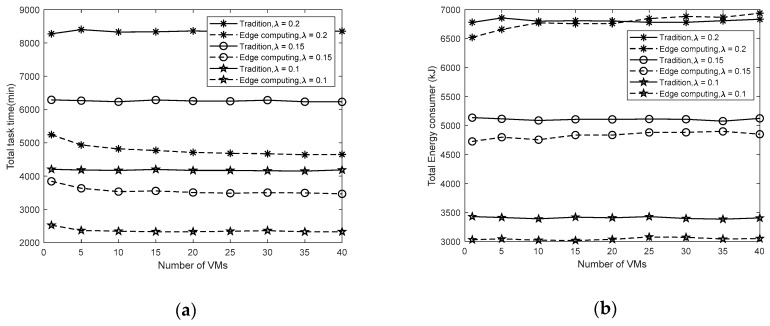
(**a**) the total time spent in terms of the number of VMs on satellite; (**b**) the total energy consumption in terms of the number of VMs on satellite.

Next, we evaluate the impact of the number of the VM on the satellite on the task time spent and energy consumption, as shown in Figure 7. In the traditional mode, the time and energy consumption of the satellite is almost independent of the number of virtual machines on the satellite. In space edge computing, the time spent decreases as the number of VMs increases and is much smaller than the time spent in the traditional mode. However, as the number of virtual machines continues to increase, time consumption will tend to be a constant value. For satellite energy consumption, it increases as the number of virtual machines increases. Furthermore, when the task generation rate of the terminal is low (*λ* = 0.1), the increase in energy consumption is not apparent. This is because the satellite performance is strong enough to meet the mission requirements. Additionally, when the task generation rate is high and the number of VMs is large, the energy consumption in the edge computing will be higher than the traditional mode. Therefore, it is not possible to reduce mission execution time by unrestrictedly increasing satellite performance, and it also leads to a sharp increase in cost. It is necessary to find a balance between time spent and energy consumption.

**Figure 8:**
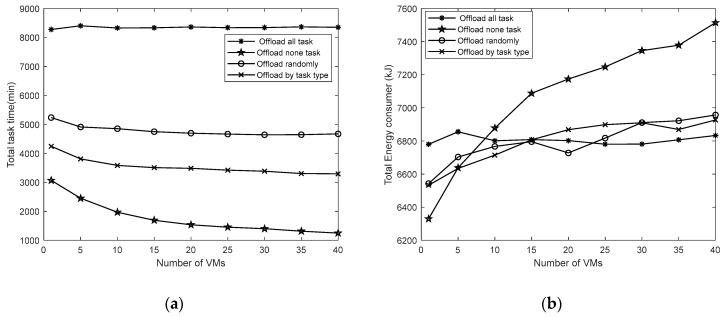
(**a**) the impact of task offloading strategy on the total time spent; (**b**) the impact of task offloading strategy on total energy consumption.

Finally, we will discuss the impact of the task offloading strategy on the time spent and energy consumption, as shown in Figure 8. Other things being equal, the task time spent is different under different offloading strategies. When the tasks are all performed on the satellite, it takes the shortest time. However, as the number of VMs on the satellite increases, the energy consumption will rise sharply. In other offloading strategies, the offloading strategy by task category takes less time than the random offloading strategy. The energy consumption of the two is similar. Therefore, for different task scenarios, the task offloading strategy is also one of the crucial factors that affects the quality of service.

## 6. Conclusions

In this article, we propose to transform traditional satellites into space edge computing nodes. The software and hardware architecture of iSat is illustrated. In terms of hardware, the original satellite hardware modules are broken up and reorganized to form various resource networks, which provides a basis for the unified management and flexible scheduling. In terms of software, edge computing technology is combined with traditional satellite software architecture to realize the virtualization of satellite resources, the flexible orchestration of satellite missions, the on-demand allocation of on-board resources and the coordinated service of satellite and ground. Furthermore, we combine the mobile edge computing and satellite orbit to establish a system model of the application scenario and analyze the execution time and energy consumption of the task. Through the simulation experiments, the space edge computing system takes less time and energy than traditional satellite constellation. The quality of service is related to the number of satellites, the performance of the satellite itself, and the satellite mission offloading strategy. In the future, we will optimize the design of the system in response to the above three aspects.

## Figures and Tables

**Table 1:** The parameters of the satellite constellation.

Parameters	Value
Number of Planes	6
Number of Satellite per Planes	11
Semimajor Axis	7159.14 km
Inclination	86.4°
